# Supplementary figures and images for: Development of an in vitro pre-mRNA splicing assay using plant nuclear extract
Source: Plant Methods. 2018 Jan 8;14:1. doi: 10.1186/s13007-017-0271-6 (PMC5757305; doi:10.1186/s13007-017-0271-6)

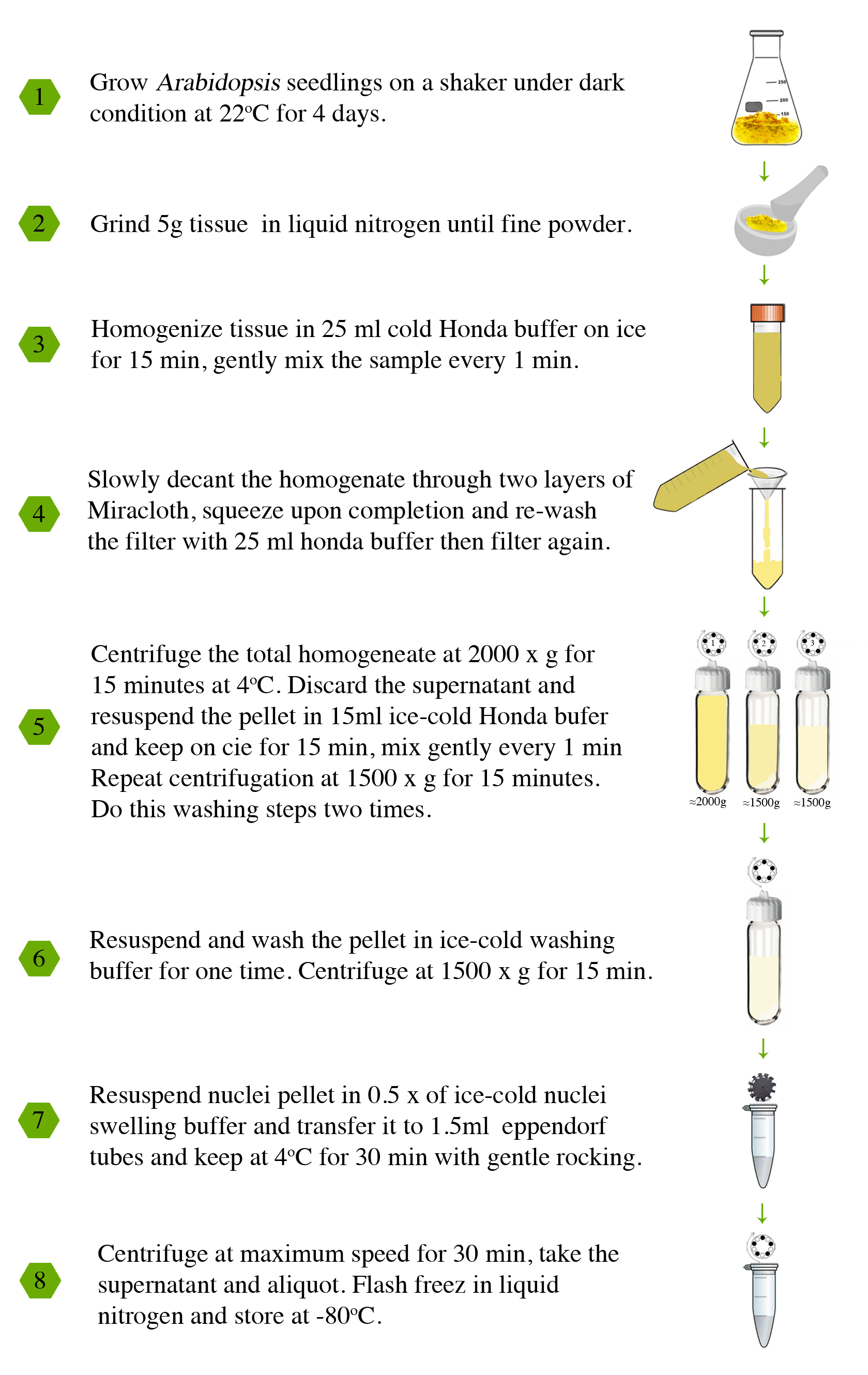

Supplement: Supplementary file 1 — Additional file 1: Figure S1. Schematic diagram showing the steps in NE preparation from four-day-old Arabidopsis thaliana etiolated seedlings. See “Methods” for details. [file 13007_2017_271_MOESM1_ESM.jpg]

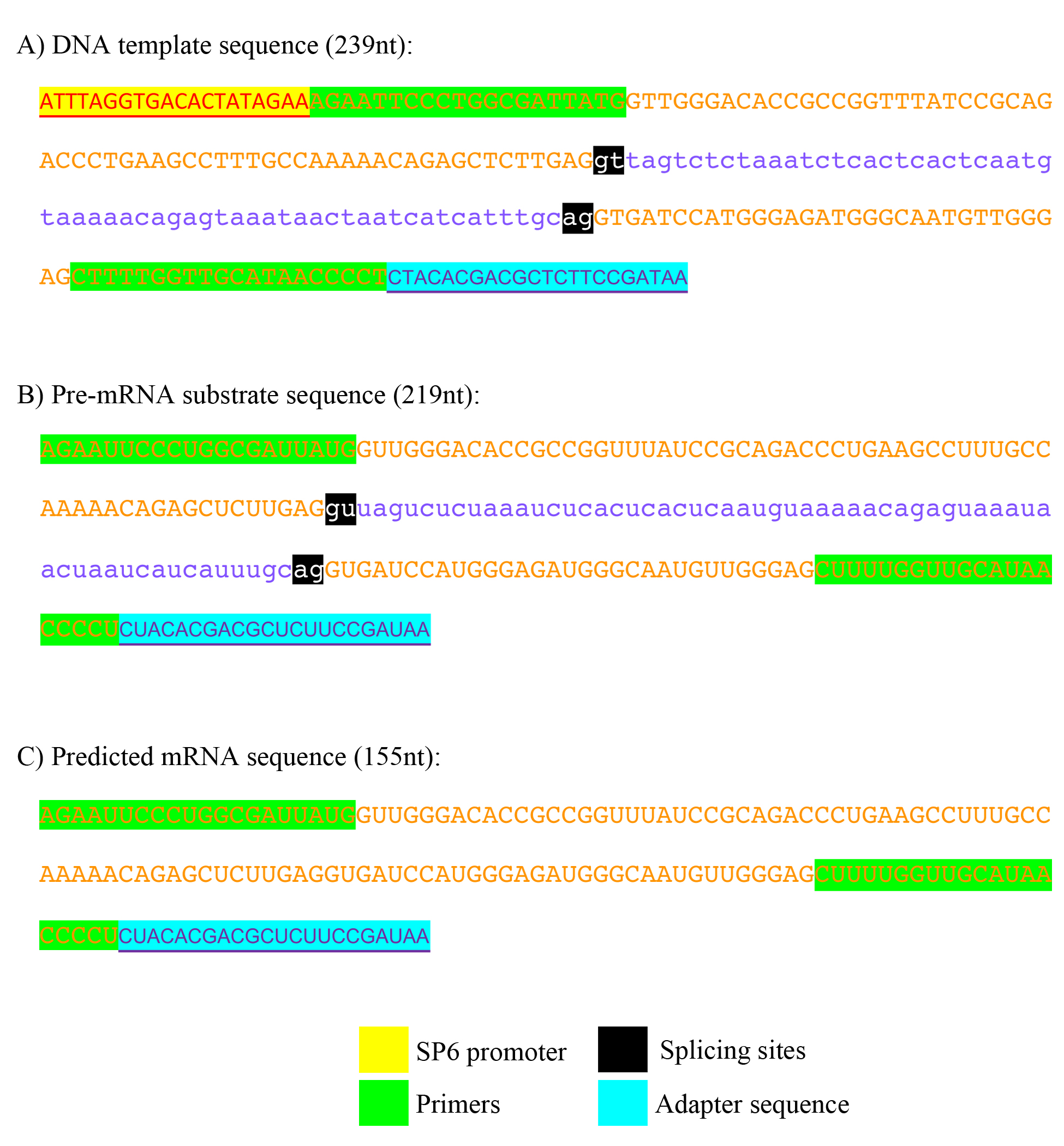

Supplement: Supplementary file 2 — Additional file 2: Figure S2. Sequences of DNA template (A), pre-mRNA substrate (B), and predicted mRNA (C). Arabidopsis LHCB3 (AT5G54270) sequence was extracted from The Arabidopsis Information Resource (TAIR). Exonic sequences are shown in upper case letters, while the intron sequence is shown in lower case. SP6 promoter sequence is highlighted in yellow; primers are highlighted in green with either SP6 or adapter sequences: conserved splicing sites (GT–AG) are highlighted in black, and adaptor sequence is highlighted in blue. [file 13007_2017_271_MOESM2_ESM.jpg]

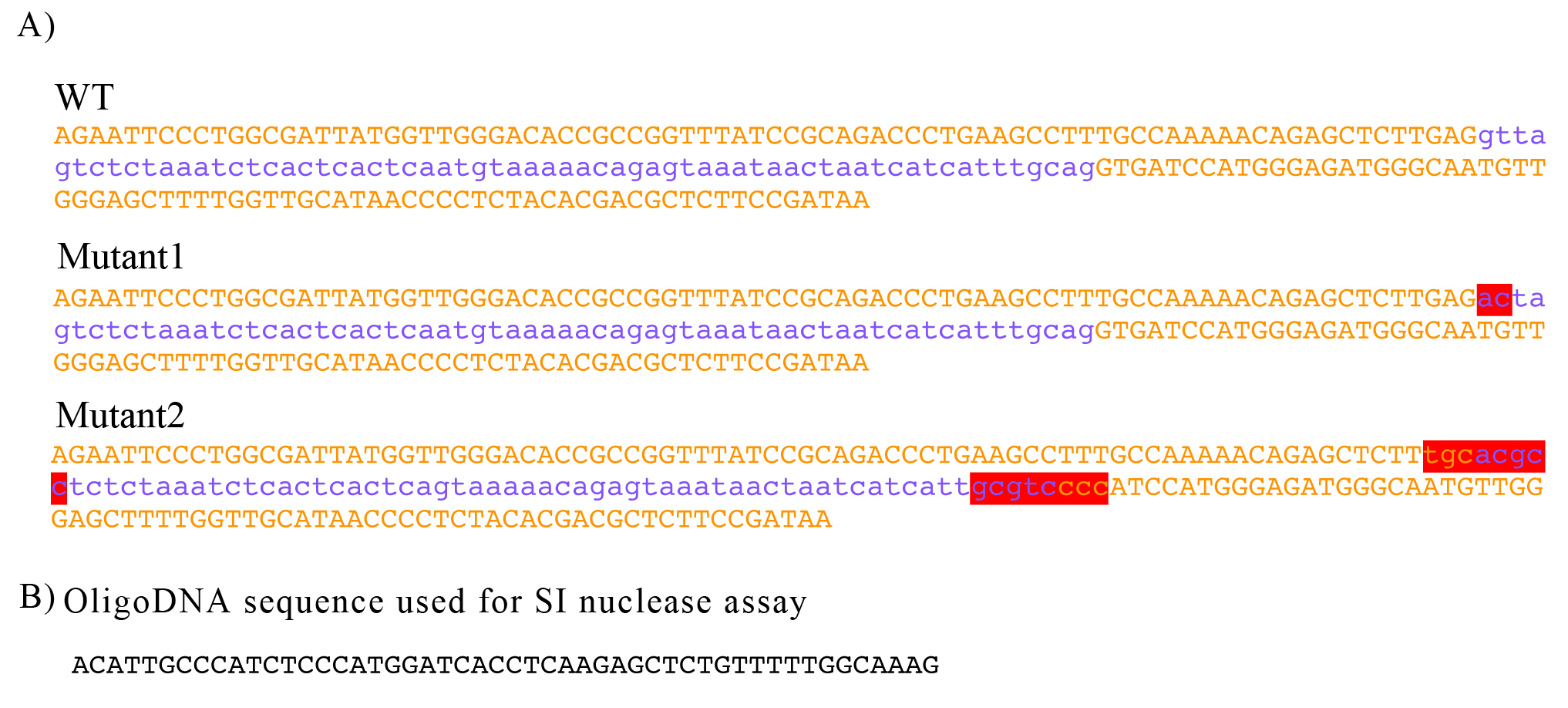

Supplement: Supplementary file 3 — Additional file 3: Figure S3. (A) Sequences of wild type (WT) and mutated (mutant 1 and mutant 2) DNA templates used to prepare pre-mRNA substrates. (B) Oligo DNA sequence used for S1 nuclease assay. [file 13007_2017_271_MOESM3_ESM.jpg]

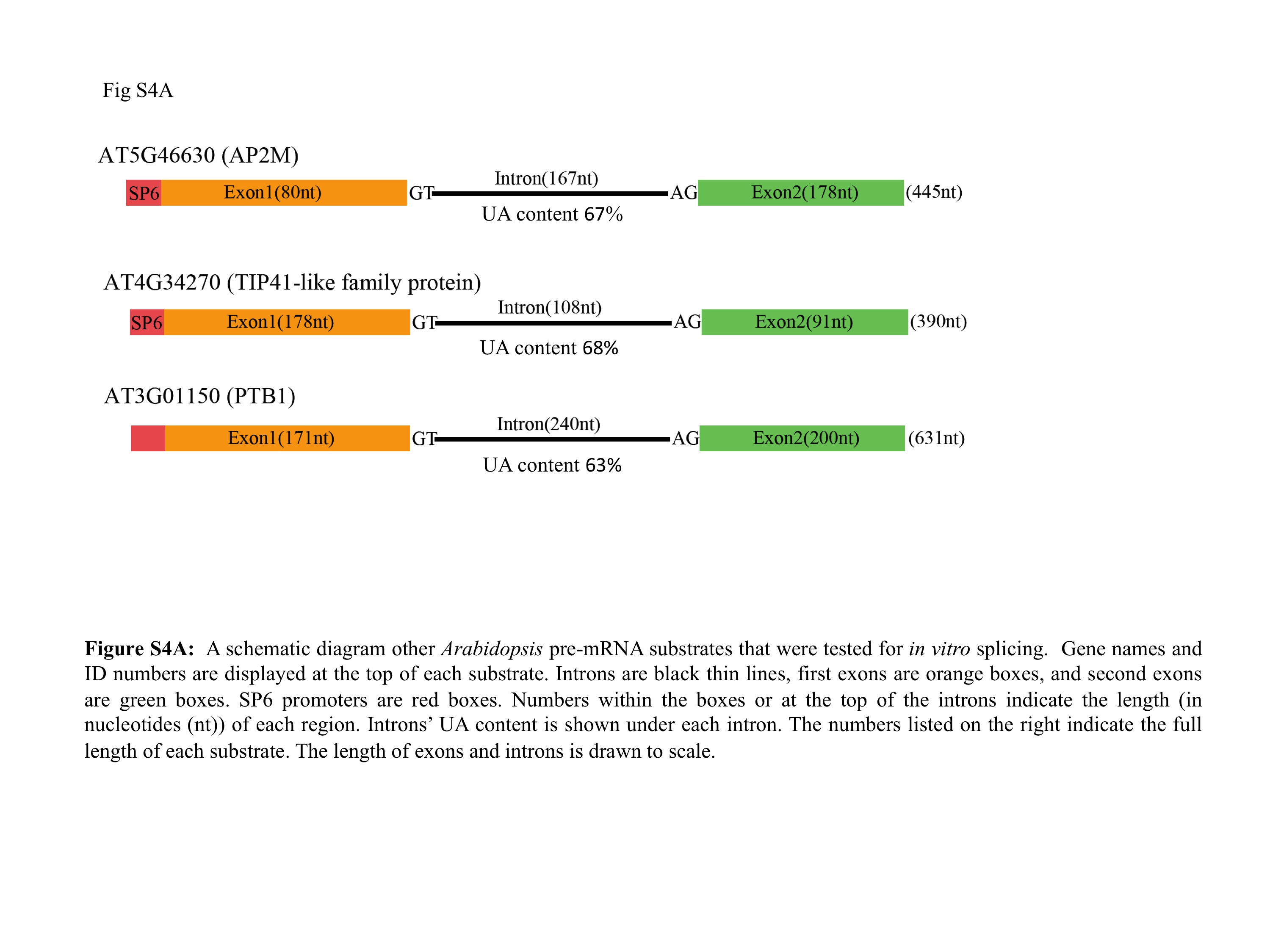

Supplement: Supplementary file 4 — Additional file 4: Figure S4. (A) A schematic diagram other Arabidopsis pre-mRNA substrates that were tested for in vitro splicing. Gene names and ID numbers are displayed at the top of each substrate. Introns are black thin lines, first exons are orange boxes, and second exons are green boxes. SP6 promoters are red boxes. Numbers within the boxes or at the top of the introns indicate the length (in nucleotides (nt)) of each region. Introns’ UA content is shown under each intron. The numbers listed on the right indicate the full length of each substrate. The length of exons and introns is drawn to scale. [file 13007_2017_271_MOESM4_ESM.jpg]

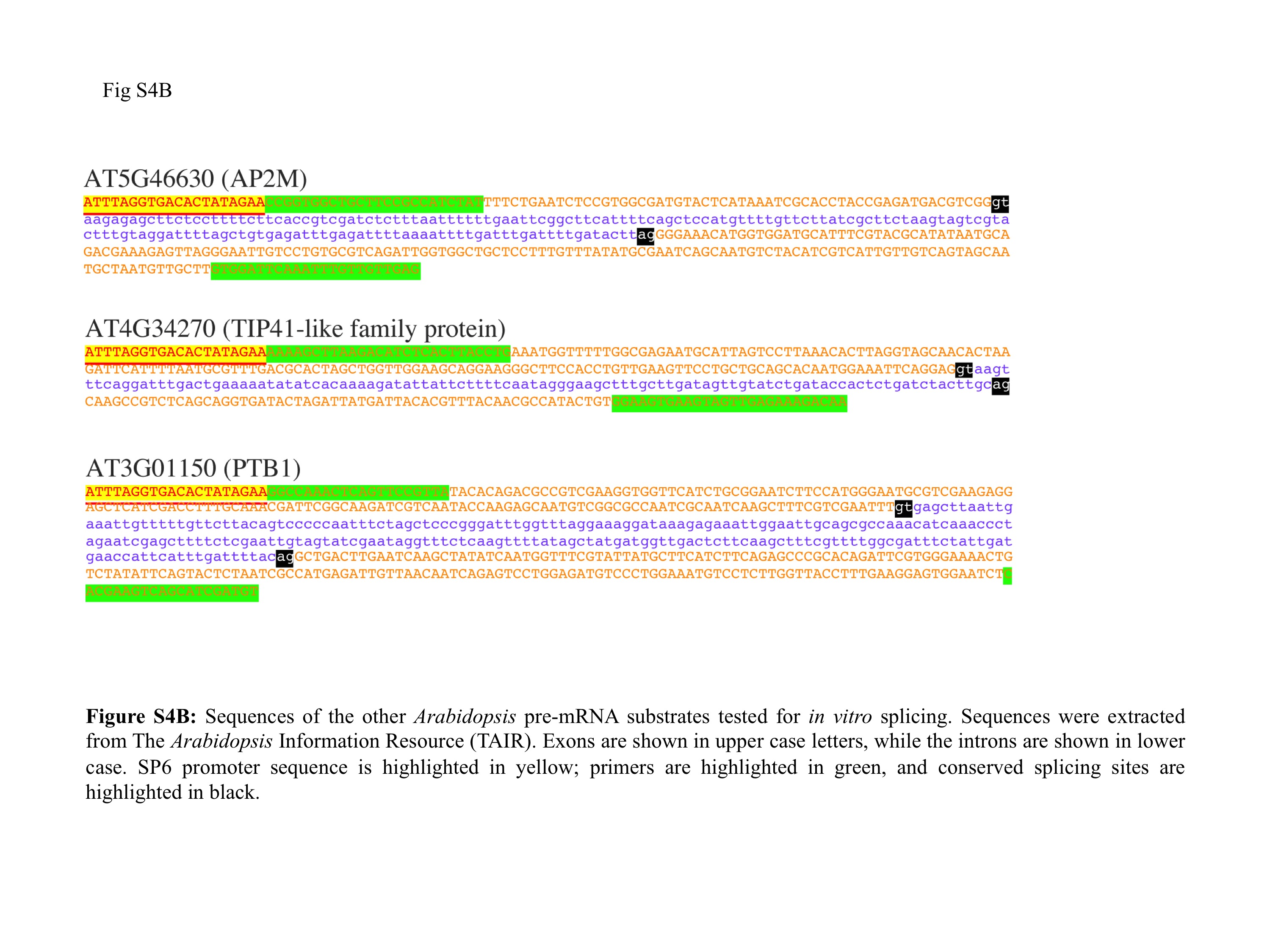

Supplement: Supplementary file 5 — Additional file 5: Figure S4. (B) Sequences of the other Arabidopsis pre-mRNA substrates tested for in vitro splicing. Sequences were extracted from The Arabidopsis Information Resource (TAIR). Exons are shown in upper case letters, while the introns are shown in lower case. SP6 promoter sequence is highlighted in yellow; primers are highlighted in green, and conserved splicing sites are highlighted in black. [file 13007_2017_271_MOESM5_ESM.jpg]

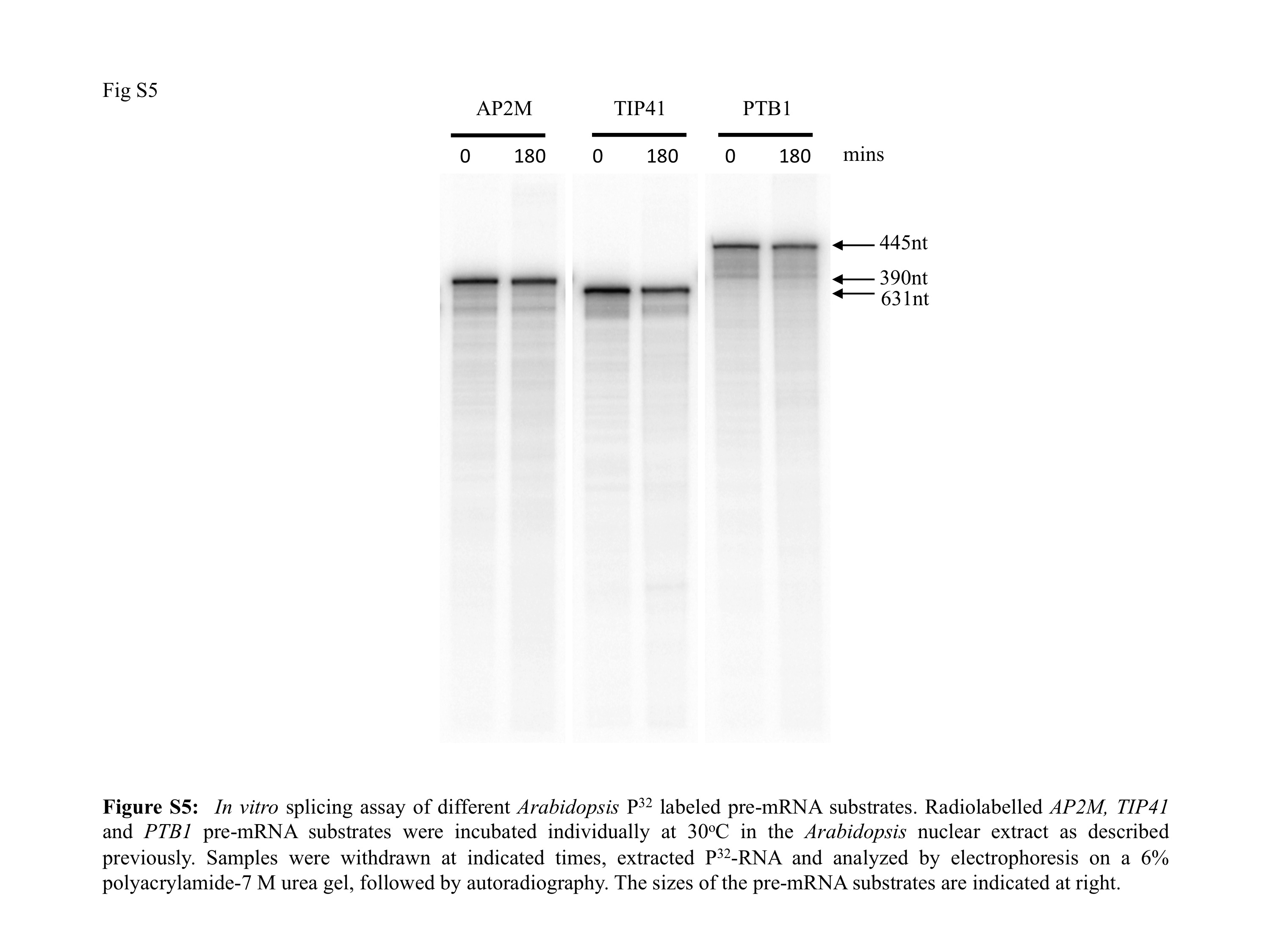

Supplement: Supplementary file 6 — Additional file 6: Figure S5. In vitro splicing assay of different Arabidopsis [32P] labeled pre-mRNA substrates. Radiolabelled AP2M, TIP41 and PTB1 pre-mRNA substrates were incubated individually at 30 °C in the Arabidopsis nuclear extract as described previously. Samples were withdrawn at indicated times, extracted [32P]-RNA and analyzed by electrophoresis on a 6% polyacrylamide-7 M urea gel, followed by autoradiography. The sizes of the pre-mRNA substrates are indicated at right. [file 13007_2017_271_MOESM6_ESM.jpg]

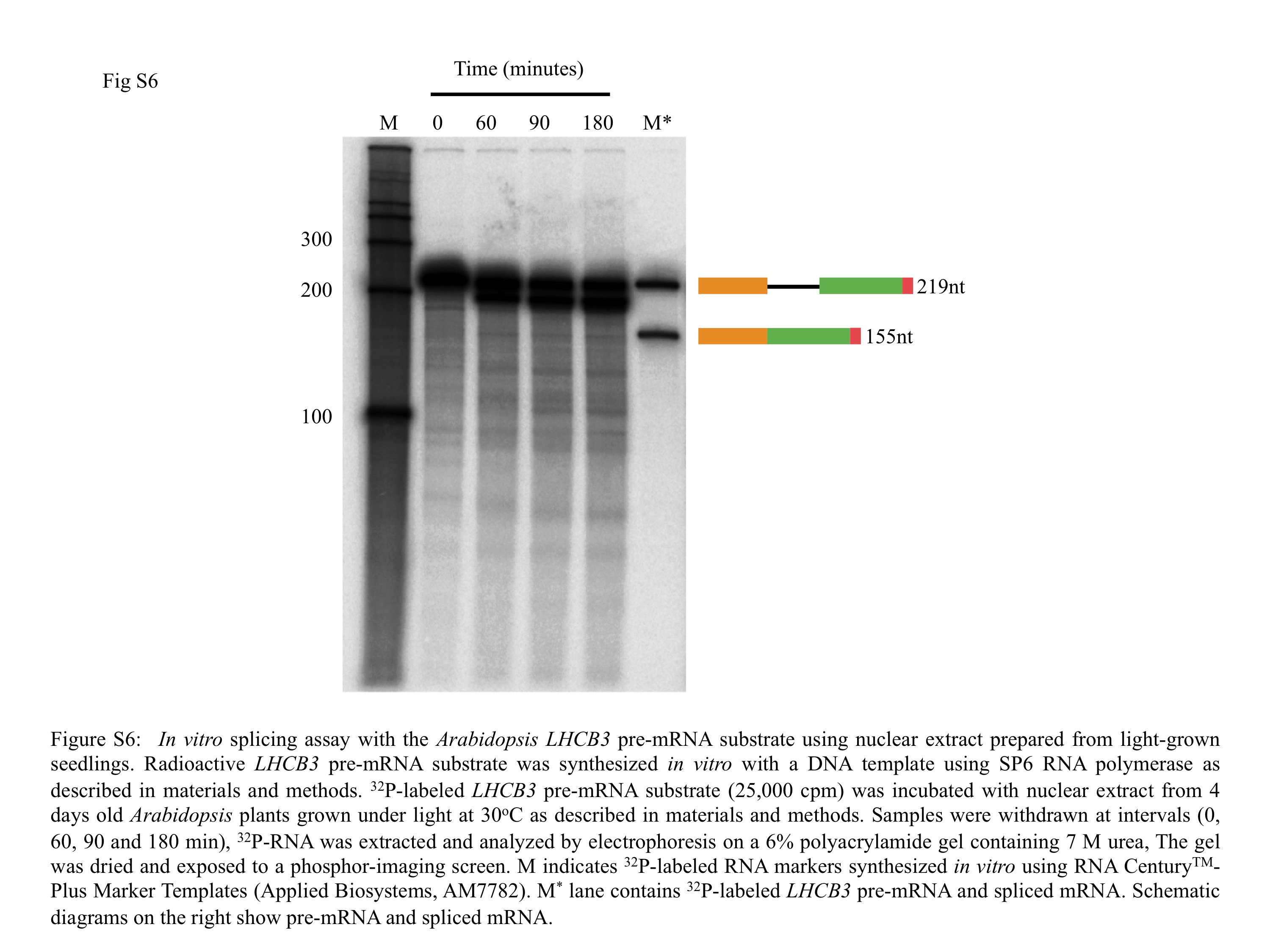

Supplement: Supplementary file 7 — Additional file 7: Figure S6. In vitro splicing assay with the Arabidopsis LHCB3 pre-mRNA substrate using nuclear extract prepared from light-grown seedlings. Radioactive LHCB3 pre-mRNA substrate was synthesized in vitro with a DNA template using SP6 RNA polymerase as described in materials and methods. [32P]-labeled LHCB3 pre-mRNA substrate (25,000 cpm) was incubated with nuclear extract from 4 days old Arabidopsis plants grown under light at 30 °C as described in materials and methods. Samples were withdrawn at intervals (0, 60, 90 and 180 min), [32P]-RNA was extracted and analyzed by electrophoresis on a 6% polyacrylamide gel containing 7 M urea, The gel was dried and exposed to a phosphor-imaging screen. M indicates [32P]-labeled RNA markers synthesized in vitro using RNA Century™-Plus Marker Templates (Applied Biosystems, AM7782). M* lane contains [32P]-labeled LHCB3 pre-mRNA and spliced mRNA. Schematic diagrams on the right show pre-mRNA and spliced mRNA. [file 13007_2017_271_MOESM7_ESM.jpg]
